# Canthaxanthin, a Red-Hot Carotenoid: Applications, Synthesis, and Biosynthetic Evolution

**DOI:** 10.3390/plants9081039

**Published:** 2020-08-15

**Authors:** Bárbara A. Rebelo, Sara Farrona, M. Rita Ventura, Rita Abranches

**Affiliations:** 1 Plant Cell Biology Laboratory, Instituto de Tecnologia Química e Biológica António Xavier (ITQB NOVA), Universidade Nova de Lisboa, 2780-157 Oeiras, Portugal; brebelo@itqb.unl.pt; 2 Bioorganic Chemistry Laboratory, Instituto de Tecnologia Química e Biológica António Xavier (ITQB NOVA), Universidade Nova de Lisboa, 2780-157 Oeiras, Portugal; rventura@itqb.unl.pt; 3 Plant and AgriBiosciences Centre, Ryan Institute, NUI Galway, H19 TK33 Galway, Ireland; sara.farrona@nuigalway.ie

**Keywords:** canthaxanthin, metabolic engineering, carotenoid biosynthesis pathway, plant secondary metabolite, chemical synthesis

## Abstract

Carotenoids are a class of pigments with a biological role in light capture and antioxidant activities. High value ketocarotenoids, such as astaxanthin and canthaxanthin, are highly appealing for applications in human nutraceutical, cosmetic, and animal feed industries due to their color- and health-related properties. In this review, recent advances in metabolic engineering and synthetic biology towards the production of ketocarotenoids, in particular the red-orange canthaxanthin, are highlighted. Also reviewed and discussed are the properties of canthaxanthin, its natural producers, and various strategies for its chemical synthesis. We review the de novo synthesis of canthaxanthin and the functional β-carotene ketolase enzyme across organisms, supported by a protein-sequence-based phylogenetic analysis. Various possible modifications of the carotenoid biosynthesis pathway and the present sustainable cost-effective alternative platforms for ketocarotenoids biosynthesis are also discussed.

## 1. Introduction: Characterization and Biosynthesis of Canthaxanthin

Carotenoids are a major class of pigments that are found in the chloroplasts or chromoplasts of photosynthetic organisms, such as plants and algae, and in some non-photosynthetic bacteria and fungi [1,2]. They are important secondary metabolites for living systems because of their protective action against photooxidative damage by intensive light in photosynthetic organisms and of their anti-oxidant properties [3,4,5,6]. Carotenoids are traditionally used as natural food colorants, cosmetics, phytonutrients, and animal feed supplements, since animals and humans do not have pathways for their de novo biosynthesis [1,2,7].

**Figure 1:**
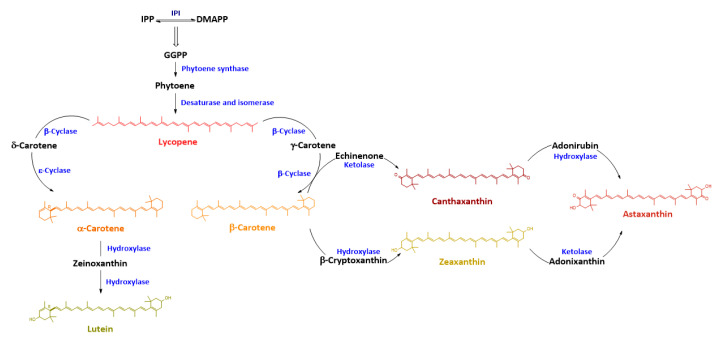
Biosynthetic pathway of phytoene-based C40 carotenoid backbones. Arrows indicate each catalyzed reaction, and enzymes are indicated in blue.

There are hundreds of carotenoids derived from the 40-carbon isoprenoid skeleton of phytoene which are mainly classified into two subgroups based on their structure: (1) carotenes, such as lycopene and β-carotene, are pure hydrocarbons, either linear or cyclized at one or both ends of the molecule, and are enriched in photosystem reaction centers; (2) xanthophylls, such as zeaxanthin and neoxanthin, contain oxygen and are most abundant in light harvesting complexes [1,2,8] (Figure 1). The xanthophyll canthaxanthin, in the same way as astaxanthin, can also be denominated as a ketocarotenoid. Ketocarotenoids are characterized by the presence of one or more ketone groups, a functional group in which an sp2 hybridized carbon atom is bound to an oxygen atom.

**Figure 2:**
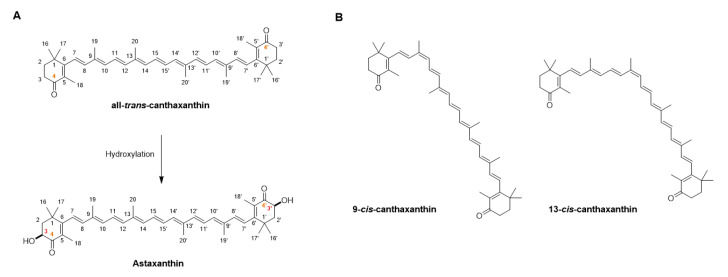
Chemical structure of (**A**) all-*trans*-canthaxanthin and astaxanthin, with assigned atom numbers; (**B**) chemical structures of two possible geometric isomers of canthaxanthin. Adapted from [11].

The pigment canthaxanthin (C_40_H_52_O_2_, 564.8 g/mol, IUPAC name: 2,4,4-trimethyl-3-[(1E,3E,5E,7E,9E,11E,13E,15E,17E)-3,7,12,16-tetramethyl-18-(2,6,6-trimethyl-3-oxocyclohexen-1-yl)octadeca-1,3,5,7,9,11,13,15,17-nonaenyl] cyclohex-2-en-1-one) is structurally represented in Figure 2. This di-ketocarotenoid, also known as β,β-carotene-4,4′-dione, has nine conjugated double-bonds, terminated by two oxo substituents at positions 4 and 4′ of the β-ionone backbone. These structural features define its characteristic red-orange color. This extended polyenone serves as a light-absorbing chromophore to shift the absorption maximum to higher wavelengths into the same region as the acyclic lycopene [9,10], known for its deep red color.

In nature, the thermodynamically more stable all-*trans*-canthaxanthin isomer is mainly observed in carotenoid extracts. *Cis*-double bonds, present in the *cis*-canthaxanthins, create greater steric hindrance between nearby hydrogen atoms or methyl groups, resulting in their lower stability. Nonetheless, conditions of temperature, acidity, or light can promote isomerization of the *trans*- to the *cis*-forms, resulting in 9-*cis*- and 13-*cis*-canthaxanthin [9,11] (Figure 2) and 13 more *cis-*isomers that have been identified [12]. The effect of isomerization has been investigated, in particular the various apoptotic and antioxidant activities of each isomer [9].

The biosynthesis of the carotenoids has been extensively studied [1,6,13] and is schematically represented in Figure 1. The 2-C-methyl-D-erythritol 4-phosphate (MEP) cytoplasmic pathway and mevalonic acid (MVA) plastid pathway are responsible for the production of isopentenyl diphosphate (IPP) and dimethylallyl diphosphate (DMAPP), the building blocks for carotenogenesis. Reactions catalyzed by phytoene synthase produce phytoene, which, by desaturation and isomerization processes, results in lycopene. Lycopene cyclases (β and ε) are responsible for generating carotenoid diversity by producing β-carotene and α-carotene. Further reactions occur via hydroxylation and ketolation [1,2]. Canthaxanthin is biosynthesized from the precursor, β-carotene, through the action of a β-carotene ketolase enzyme (BKT in algae and CRTW in bacteria). The enzyme oxidizes the allylic 4-position to a carbonyl group in the β-ring, producing the intermediate echinenone. Sequentially, the same enzyme transforms the 4′-carbon atom in the second β-ring to a carbonyl [14].

Canthaxanthin is not only a high-value product with many applications in the market, but is also the substrate for the production of another ketocarotenoid of commercial interest, astaxanthin. As depicted in Figure 2, the enzyme β-carotene hydroxylase introduces hydroxyl moieties into the canthaxanthin rings at 3,3′-positions, resulting in the formation of astaxanthin [15]. These carotenoid hydroxylases are differently denominated according to the species: CHY, HYDB, BCH, or BHY for plants and algae and CRTZ for bacteria [1].

**Figure 3:**
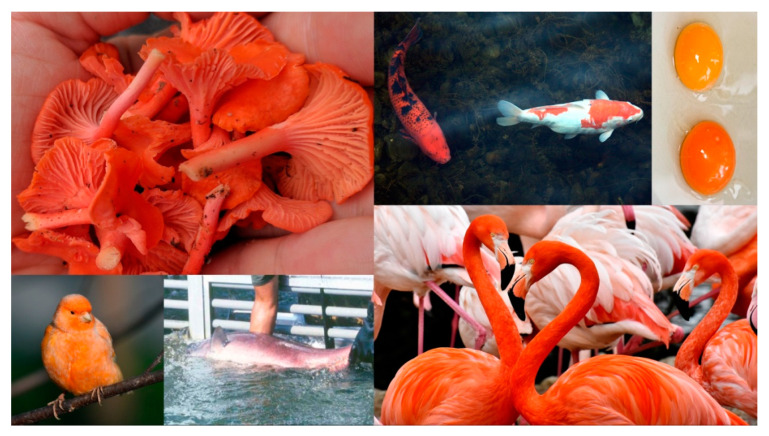
Reddish-orange coloration present in fauna, flora, and food derivatives due to ketocarotenoids. Clockwise: edible mushroom *Cantharellus cinnabarinus*, Koi carps, egg yolk, flamingo, salmon from aquaculture, and canary.

Amongst the hundreds of carotenoids identified, canthaxanthin belongs to the few that are marketed commercially with a wide range of applications. There is a commitment to provide consumers with more “colorful” and “healthier” foods [16,17]. The red-orange color in numerous animals is derived from their diet [1,2,7,13], indirectly either by the ingestion of carotenoid-containing organisms or by the consumption of feed supplements enriched with carotenoids. Coloration caused by canthaxanthin can be observed in flamingo feathers, koi carp skin, salmon and trout flesh, and egg yolks (Figure 3) [16,18,19].

Health-related benefits associated with carotenoid intake have been extensively reviewed [6,8,13,16,20,21]. Here, we highlight some examples focused on canthaxanthin studies. Due to its lipophilic nature, canthaxanthin can accumulate in the fatty tissues of food [16] when consumed by animals. In humans, these carotenoids are released from the food matrix and incorporated into mixed micelles made of bile acid, fatty acids, and lipids. The amount of each carotenoid incorporated into micelles depends on the micellar fatty acid composition and the polarity of the carotenoid [20]. Xanthophylls display a higher bioavailability than carotenes, probably because hydroxyl groups may increase their solubility in the micellar structures. Uptake efficiency seems to correlate with carotenoid polarity and flexibility, since polar and flexible carotenoids have a better affinity for lipid transporters and/or plasma membranes, which would lead to an increased absorption [22]. The absorption, transport, and bioavailability processes for carotenoids can be found in more detail in [8,16,20,21,22].

Carotenoids, or their degradation metabolites, may have a particular role in inflammation-related processes by (1) activation of nuclear factor erythroid 2-related factor 2 (Nrf2)-dependent oxidant response or (2) suppression of nuclear factor кB (NF-кB). Briefly, Nrf2 is a redox-sensitive transcription factor “master regulator” of cell survival by counteracting oxidative stress and modulating redox signaling events. NF-кB consists of a family of transcription factors that regulate a large array of genes involved in inflammatory and immune responses. More detail about these transcription factors and how carotenoids can elicit these signaling pathways can be found in [13,23,24,25]. Furthermore, carotenoids play a significant role in the redox metabolism due to their prooxidant activity and/or antioxidant activity by scavenging and quenching reactive species [13,25]. These can have damaging roles as powerful oxidants, for example by targeting lipids and affecting key regulatory proteins, such as the Nrf2 complex. On the other hand, they contribute to maintaining the central homeostatic mechanisms key for cell survival through redox signaling. This cellular pathway may affect gene transcription, enzyme activity, and membrane and genome integrity [26].

Canthaxanthin, being a ketocarotenoid, exhibits higher antioxidant and free radical scavenging properties than other carotenes and xanthophylls. Studies have shown that canthaxanthin is capable of scavenging reactive oxygen species and quenching singlet oxygen [9,27]. In addition, in vivo experiments revealed that canthaxanthin supplementation led to a decrease in lipid peroxidation to prevent induced liver DNA damage in rats [28] and to enhance the antioxidant defense in rat liver [29,30]. At the immunomodulatory level, an interesting example was observed in a mouse cell line, in which canthaxanthin increased the activity of alkaline phosphatase, an enzyme found in several tissues involved in the detoxification of lipopolysaccharides. Further studies are necessary to evaluate the role of canthaxanthin in mitigation of the damaging effects of lipopolysaccharides in infectious diseases and chronic inflammation [16]. Regarding the uses of canthaxanthin in humans, there are no comprehensive studies about the effects and benefits. Currently, canthaxanthin is popularly used as a natural skin-tanning agent, used in cosmetics, and which, if used in large quantities, produce an orange-brown color to the skin [31]. Canthaxanthin, with its many properties, may positively affect the quality of animal-derived food products and feed supplements and the health status of both human and animal consumers.

## 2. Economic Value of Ketocarotenoids and Market Potential

The increasing consumption of carotenoid-containing products is driving the market to grow. A recent in-depth market analysis on the topic revealed that the global carotenoids market was valued at $1577 million in 2017 and is expected to reach $2 billion by 2022 [32,33]. In 2017, the canthaxanthin market size reached $75 million, with 40% of volume share accounted for animal feed applications [34,35]. This pigment is projected to exhibit a compound annual growth rate of 2.2% by 2024, resulting in a market size of approximately $85 million [34,35]. Canthaxanthin has been licensed in over 70 countries in Europe, America, and Asia [34,35]. The benefits of dietary carotenoids in health are encouraging the shift of commercial production of canthaxanthin from chemical synthesis to consumption or extraction from natural sources [13].

Carotenoid denominations often give hints as to the natural sources in which they were found, as is the case of canthaxanthin. This pigment was first isolated from an edible chanterelle mushroom, *Cantharellus cinnabarinus* (see Figure 3, Section 1), as a major component in a mixture of carotenoid extracts [36]. Since then, canthaxanthin has been identified in several species. Table 1 depicts the most relevant natural source of ketocarotenoids.

Natural production systems, such as bacteria, algae, or plants, are being explored in the search for a cost-effective production of ketocarotenoids at high yields that are safe for humans and environmentally-friendly [9,63]. Several efforts have been reported to improve large-scale production of high-value carotenoids, e.g., downregulation of the carotenoid divergent pathway, improving carotenoid storage capacity, or overexpressing rate-limiting enzymes [13]. Moreover, the market may also benefit from the advances in synthetic biology and biotechnological tools towards industrial production of natural carotenoids [33,34]. This will be reviewed with more detail in Section 5.

## 3. Chemical Synthesis of Canthaxanthin, the Main Approach

Currently, several efforts are being made to improve the large-scale production of carotenoids to accommodate the ever-increasing demand, and these include the optimization of canthaxanthin chemical synthesis, since it is the main source of production for the market of animal feed supplements [9,63]. Here, we describe three methodologies for the chemical synthesis of all-*trans* canthaxanthin.

**Scheme 1:**
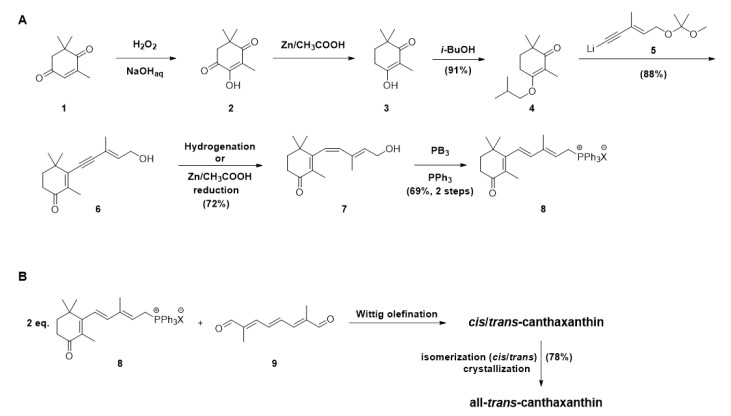
Scheme of the synthesis of canthaxanthin, according to [16,64,65]. Preparation of C_15_-Wittig salt (**A**) and all-*trans*-canthaxanthin (**B**).

The first synthesis of canthaxanthin is shown in Scheme 1. 6-oxo-isophorone was converted after several steps into the corresponding C_15_-Wittig salt 8 (Scheme 1), which is a key intermediate for the canthaxanthin synthesis [16,64]. *Z*/*E*-canthaxanthin was obtained via a double Wittig olefination by mixing two equivalents of compound 8 and the symmetrical building block C_10_-dialdehyde 9. The two isomers can be separated by crystallization, since the all-*E*-canthaxanthin is poorly soluble [16,64,65]. The main drawback of this Wittig olefination is the formation of triphenylphosphine oxide as a by-product, which makes the reaction atom-inefficient, and it is also difficult to separate from the desired reaction product [16].

**Scheme 2:**
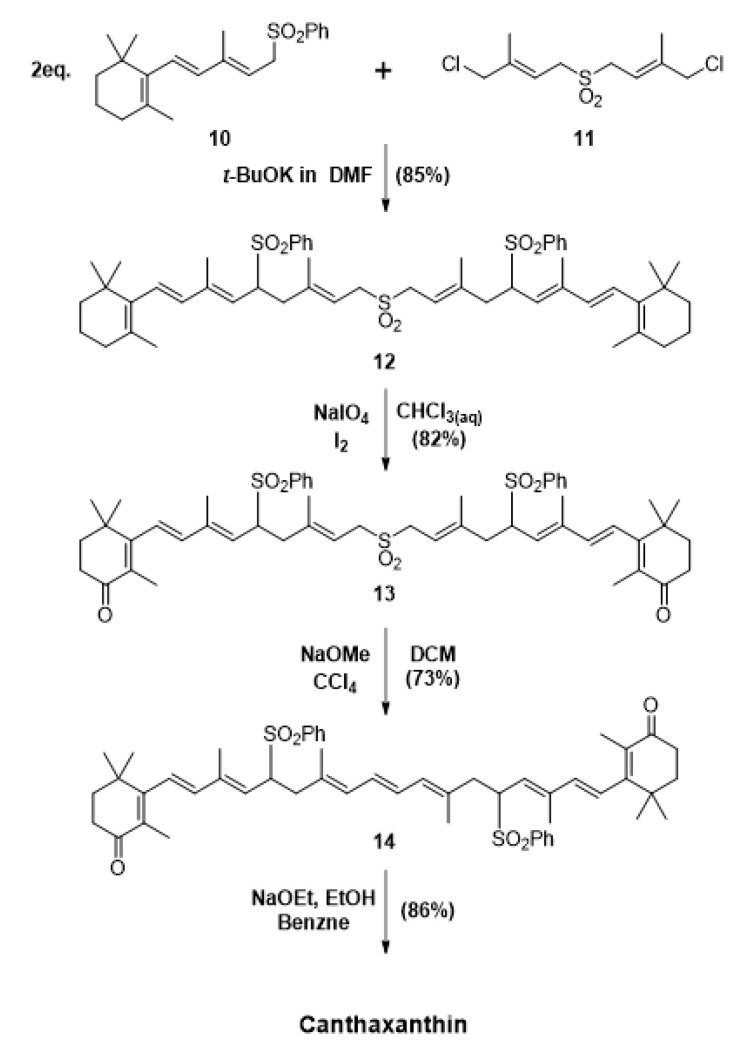
Scheme of canthaxanthin synthesis via C40-trisulfone. Adapted from [66].

Another synthetic route was developed using sulfone intermediates, thus avoiding the use of triphenylphosphine, as outlined in Scheme 2. The coupling reaction of one equivalent of C_10_-bischloroallylic sulfone 11 with two equivalents of C_15_-allylic sulfone 10 afforded C_40_-trisulfone 12. Compound 13 was then obtained by iodine-catalyzed allylic oxidation at the cyclohexene rings of compound 12. The Ramberg–Bäcklund reaction of the oxidized trisulfone 13 under Meyer’s conditions gave the desired C_40_-disulfone 14. The base-promoted dehydrosulfonation with sodium ethoxide in ethanol of compound 14 provided the final product, canthaxanthin [16,66].

**Scheme 3:**
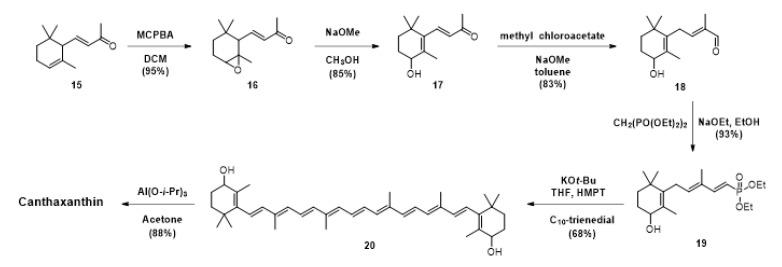
Synthesis of canthaxanthin according to [67].

Very recently, a novel route for the total synthesis of canthaxanthin was described by Pi et al. ([67] Scheme 3). Epoxidation of α-ionone 15 with metachloroperbenzoic acid afforded the epoxide 16, which later was converted to 4-hydroxy-β-ionone 17 in the presence of sodium methoxide. Next, the C_14_-aldehyde 18 was obtained by a Darzens condensation between methyl chloroacetate and compound 17, followed by a decarboxylation reaction. A Wittig–Horner condensation with tetraethyl methylenebisphosphonate and compound 18 was carried out to afford C_15_-phosphonate 19, which was then condensed with C_10_-trienedial 9 in the presence of potassium *tert*-butoxide, affording compound 20. Under these reaction conditions, isomerization of the 1,3-diene in 19 to the required 2,4-diene occurred. Finally, an Oppenauer oxidation of the hydroxyl groups of 20 catalyzed by aluminium isopropoxide resulted in the target product, canthaxanthin [67]. The resulting crude canthaxanthin contained 15–20% *cis*-isomer, thus an isomerization of the crude product was performed by refluxing it in isobutanol for 10 h, reducing the content of the *cis*-isomer to 3%.

## 4. Evolution of Canthaxanthin Biosynthesis

Whilst the biosynthesis and structure of carotenoids have been studied extensively at chemical and molecular levels, much less is known about the evolution of the enzymes of the pathway. Owing to the acquisition of key enzymes, various organisms can produce specific combinations of pigments as a result of the diversification of the biosynthetic carotenoid pathway. Nevertheless, there are questions that remain unanswered: When did specific carotenoid biosynthetic enzymes appear during evolution? How were their genes conserved throughout the tree of life? Future research in the evolution of the enzymes of the carotenoid pathway will be paramount for precise modulation of pigment synthesis.

Phylogenetic analyses, together with the use of mutants, have so far contributed to our understanding of the functional overlaps for different steps of the carotenoid pathway [68,69]. An elegant combination of classical interspecific evolutionary approaches—based on protein sequence comparison—and more advanced intraspecific approaches (i.e., microsatellite genotyping, sequencing of PCR products, whole-genome resequencing, and in silico translation) permitted the definition of the phylogenetic tree of a carotenoid dioxygenase enzyme (CCD4) involved in fruit flesh color and to propose a model to link phenotypic variations in peach pigmentation to the evolution of CCD4 throughout the domestication of this orchard crop [70]. Protein sequence analyses can help to prevent undesirable results in heterologous systems when expressing gene-encoding carotenoid biosynthetic enzymes. For instance, the presence of specific proteins in the selected system may modify the target pigment, decreasing its final yield or producing unexpected carotenoids [71]. The study of sequence conservation applied to molecular engineering has revealed crucial residues to control the activity of key enzymes and increase the production of carotenoids [69,72,73]. This is also relevant because the combination of carotenoids produced in an organism may not exclusively be the consequence of the presence of specific genes but of enzymatic variants [74]. A deeper research into the co-evolution of enzymes may also contribute to the understanding of why, in heterologous systems, the combinatorial overexpression of independent genes from a variety of organismal sources favors higher pigment yields in comparison to other enzymatic combinations (please see Table 2 in Section 5). An increasing catalogue of full genome sequences also allows the discovery of molecular tools to redesign endogenous carotenogenesis, which may mark a new era for synthetic evolution [75,76]. So far, most of the efforts to increase carotenoid production have largely been based on the overexpression of gene-encoding key carotenogenic enzymes. Future phylogenetic analyses of conserved *cis*-elements in the regulatory sequences of the genes encoding these enzymes, which may be able to control transcription in response to cues, such as nutrients, temperature, or light variations, will set further light on fine-tuning the reprogramming of the pathway for the breeding of novel varieties. This new direction in the study of carotenoid synthesis will also contribute to understanding the ability of species to respond to a more unpredictable environment due to climate change, which may have profound ecological consequences for carotenogenic species.

Many organisms are able to produce β-carotene, but only a few species among them can use this molecule as a substrate for the production of canthaxanthin as a final or intermediate product. This is the consequence of the acquisition of enzymes with ketolase activity. The BRENDA enzyme database (www.brenda-enzymes.org; [89,90]) classifies the β-ketolases as part of a large protein family of oxidoreductases. The ability of β-carotene ketolase enzymes to produce carbonyl groups depends on the species. For instance, CRTW-type ketolases can symmetrically introduce keto groups, simultaneously modifying both β rings, while CRTO ketolases (mostly found in cyanobacteria) modified only one of the β rings to produce echinenone (Figure 1) [91]. Lateral gene transfer was probably behind the acquisition of *crtW* and *crtO* genes, as proposed in general for the evolution of *crt* genes in bacteria, although *crt* genes seem to be more scattered across cyanobacteria than eubacteria phylogenetic trees. The genes which encode for ketolases may have later passed on algae from cyanobacteria as part of the horizontal gene transfer of the endosymbiotic process [74]. This hypothesis implies that the cyanobacteria that acted as endosymbiont were already able to produce ketocarotenoids, as has been already proposed for other key enzymes involved in earlier steps of the carotenoid biosynthesis [92]. *crtO*-like genes have also been identified in protist genomes; however, their functions and evolutionary relationships still need further elucidation [93].

**Figure 4:**
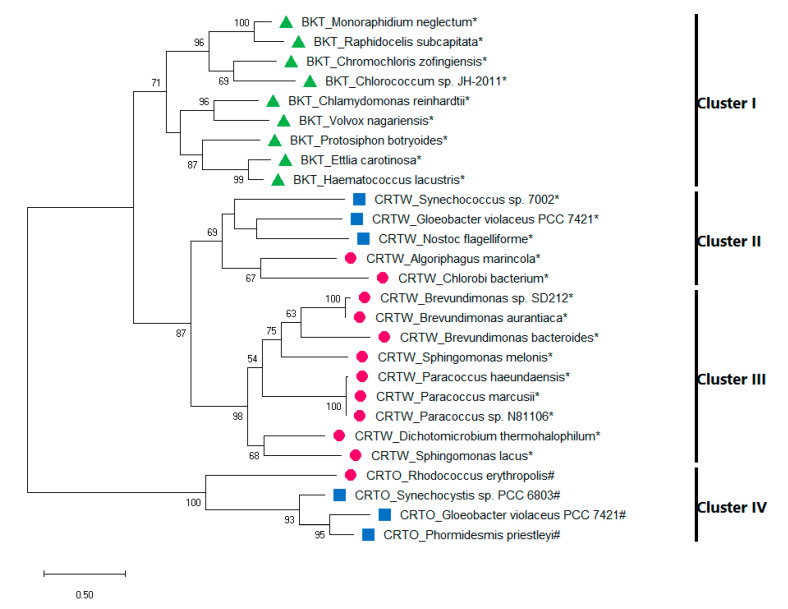
Phylogenetic analysis of β-carotene ketolase enzymes from different organisms: algae (green triangle), cyanobacteria (blue square), and bacteria (pink circle). The phylogenetic tree was built with MEGA X software by applying the maximum likelihood method. Only one protein sequence was used when there was homology of multiple sequences. Numbers associated with the branches were the maximum likelihood bootstrap values (*n* = 1000). Protein sequences with FA_desaturase (PF00487) domain and Amino_oxidase (PF01593) domain are represented with * and #, respectively. The tree is drawn to scale, with branch lengths measured in the number of substitutions per site.

To investigate the phylogenetic relationships of the enzymes that are responsible for the production of ketocarotenoids in several organisms, we carried out a phylogenetic analysis using Molecular Evolutionary Genetics Analysis (MEGA X, [94]) software based on multiple alignments of protein sequences (Figure 4). Cluster I is composed of proteins encoded by *bkt* genes from the same class of green algae, Chlorophyceae. Searching for conserved domains in these BKT proteins, using the Conserved Domain Database (CDD, https://www.ncbi.nlm.nih.gov/cdd/, [95]) and Pfam database (https://pfam.xfam.org, [96]), indicates the presence of a FA_desaturase (PF00487) domain, which correlates with a fatty acid desaturase conserved domain responsible for the insertion of a double bond in fatty acids. Cluster II is composed of proteins encoded by *crtW* genes from cyanobacteria and bacteria that also have the fatty acid conserved domain. Even though they are taxonomically different, it appears that these CRTW proteins may have a similar function in the ketocarotenoid biosynthesis, since the proteins share the same domain as the proteins from cluster I. The genome of cyanobacterium *Gloeobacter violaceus* has two ketolase genes that are homologous to other β-carotene ketolase genes, both of them annotated in the National Center for Biotechnology Information (NCBI) database as *crtO*. However, the protein sequence from this cluster, encoded by the *gll1728* gene from *G. violaceus*, presented a *crtW*-like function in a β-carotene-producing *Escherichia coli*. [97,98]. Therefore, the protein encoded by this sequence is indeed a CRTW protein. On the other hand, the green sulphur, *Chlorobi bacterium*, has two *crtO* genes annotated in NCBI. However, proteins encoded by these genes share 69% of sequence identity and have the same conserved domain (FA_desaturase). Moreover, they are clustered together with cyanobacteria and bacteria that have been described as having the CRTW-type β-carotene ketolases [99,100]. As a result, we consider the annotated CRTO sequences from *Chlorobi* to be putative CRTW proteins. Cluster III is composed of proteins encoded by *crtW* genes from bacteria, specifically from the phylum Proteobacteria. All the sequences within this cluster have the same conserved domain as the one described for cluster I (FA_desaturase). Hence, although bacteria and algae belong to distinct taxonomic domains, the enzymatic domain in the β-carotene ketolase was conserved throughout evolution. Cluster IV is composed of proteins encoded by *crtO* genes from three species of cyanobacteria, *Synechocystis* sp. PCC 6803, *G. violaceus*, and *Phormidesmis priestleyi*, and one species of bacteria, *Rhodococcus erythropolis*. While these species form a completely independent cluster, indicating that the function of the members of this cluster differs from other proteins of the family, the other three clusters seem to have evolved from a common evolutionary event. The analysis of the conserved domains of the proteins indicates that these organisms possess different conserved domains, and for this reason, this cluster is indeed an outgroup [91]. They share an Amino_oxidase domain (PF01593), characteristic for various amine oxidases, and the NAD_binding_8 domain (PF13450), which is a NAD(P)-binding Rossmann-like domain. *P. priestleyi* additionally has a FAD-dependent oxidoreductase domain (PF01266) and *Synechocystis* sp. PCC 6803 also possess a FAD-dependent oxidoreductase and belongs to the FAD binding family (PF00890). As described before, *G. violaceus* has two *crtO* annoted protein sequences that are not identical. For that reason, and the different conserved domains these proteins have, one of them is in cluster II and the protein encoded by the *gll0394* gene is in cluster IV. The novel β-carotene ketolase of *G. violaceus*-entitled *crtO* has been referred to as structurally related to the phytoene hydrogenase *crtI*-type [91]. In *G. violaceus*, phytoene is converted to lycopene by a single bacterial-type phytoene desaturase (*crtI*), whereas in other cyanobacteria phytoene desaturation is achieved by two enzymes [101].

In the higher plant *Adonis aestivalis*, an alternative metabolic process evolved to produce canthaxanthin as an intermediate pigment in the synthesis of astaxanthin. In this species, the enzyme hydroxy-β-ring 4-dehydrogenase (HBFD) requires 4-hydroxyl rings to introduce the ketone group. It has been discussed by Cunningham and Gantt that this enzyme could originally have evolved from a plastid dehydrogenase of unclear function [7]; although, considering evolutionary studies, it is most probably unrelated to carotenogenesis. A characteristic of HBFDs and related proteins in other species is the presence of a NADP binding domain, suggesting a role for this cofactor in their activity [7]. In addition, *A. aestivalis* HBFDs are shorter than other related plant sequences as they lack a final C-terminal peptide; however, the impact of the missing amino acids in their specific metabolic function is still unknown. The analysis of the sequence of HBFDs has identified related proteins in plants, algae, cyanobacteria, and several bacteria species and have also shown that, in other species, these sequences have been misidentified as saccharopine dehydrogenases (SDHs). However, HBFD and SDH enzymes diverged early during evolution [7], possibly indicating an independent mode of action. Although plant HBFDs may have originated from the cyanobacteria endosymbiont, the presence of bacterial-related sequences within this protein family indicates a more ancestral and photosynthesis-independent common precursor [7]. Further molecular evolutionary evidence is still needed to clarify the function of the HBFD family in many other species.

All these different organisms frequently do not produce canthaxanthin in sufficient quantities [9] and, therefore, cannot economically compete with its synthetic production. On the other hand, it is difficult to isolate canthaxanthin from some species, since the majority of this ketocarotenoid is the substrate for the production of astaxanthin. The lack of suitable bioproducers dictates that commercial canthaxanthin is supplied by chemical synthesis [77] but also brings the opportunity to develop improved alternative production systems.

## 5. Canthaxanthin Production in Heterologous Systems

Due to the high value and increasing demand in the nutraceutical market, several methods have been developed to produce ketocarotenoids on a large scale and in a more sustainable way as an alternative to synthetic production. As discussed, the ketolation reaction necessary to produce ketocarotenoids is restricted to a few species. However, canthaxanthin production can be achieved in heterologous systems by genetic engineering of the metabolic carotenoid biosynthesis pathway [3,13]. Metabolic engineering allows for the production of carotenoids in organisms that do not produce them naturally, as well as modulating existing carotenoid pathways. It is a powerful tool that enables the cloning of heterologous genes from different sources into a suitable production system. Genetic modification for ketocarotenoid production has been accomplished in yeast, bacteria, and plants, giving rise to a number of hydroxylated/ketolated carotenoids with variable qualitative and quantitative profiles [4], as summarized in Table 2.

Recently, Gao and coworkers [79] have generated a transgenic cyanobacteria *Nostoc* sp. PCC 7120, overexpressing a heterologous β-carotene ketolase gene from *Nostoc flagelliform*. This engineered strain had a substantial increase on canthaxanthin biosynthesis, as well as a greater production of the intermediate substrate echinenone. In bacteria, a high-titer canthaxanthin producing a *E. coli* strain was achieved by Scaife et al. [77]. They conjugated modifications to the mevalonate-independent pathway with the transfer of a β-carotene ketolase gene (*crtW*) from *Anabena variabilis*, resulting in strains capable of producing variable amounts of canthaxanthin and other carotenoid precursors. Furthermore, they introduced additional mutations to hamper the formation of byproducts or to increase glyceraldehyde 3-phosphate production. Following the same rationale, this time in a fungus, Papp et al. [88] obtained stable *Mucor circinelloides* canthaxanthin-producing strains after transformation with a β-carotene ketolase gene (*crtW*) from marine bacterium *Paracoccus* sp.

Even though only a few papers have reported canthaxanthin production as the main goal, this ketocarotenoid was also produced in quantifiable amounts in higher plants. Carotenogenesis can be genetically manipulated towards heterologous astaxanthin biosynthesis and, at times, it was found that the transgenic astaxanthin-producing plants were also yielding the precursor canthaxanthin. Canthaxanthin accumulation was dependent on the type of tissue and on the endogenous β-carotene hydroxylase activity, since it is responsible for the conversion of canthaxanthin into astaxanthin. Huang and colleagues [85] expressed the algal β-carotene ketolase gene (*bkt*) from *Chlamydomonas reinhardtii* and the β-carotene hydroxylase gene (*bhy*) from *H. pluvialis* in tomato, leading to appreciable accumulation of ketocarotenoids. The transgenic lines, expressing only *bkt*, accumulate canthaxanthin as a major ketocarotenoid. Similar results were obtained, also in tomato, by Enfissi et al. [102] with the insertion of β-carotene hydroxylase and ketolase genes (*crtZ* and *crtW*) from the bacteria *Brevundimonas* sp. In another study, Zhu and colleagues transformed rice endosperm with multiple carotenogenic genes [82]. They combined the *bkt* gene from *C. reinhardtii*, the *bhy* gene from *H. pluvialis*, and phytoene synthase- and phytoene desaturase-coding genes from maize and bacteria *Pantoea*, respectively. The metabolic manipulation resulted in various types of germplasm, from the yellow-grained Golden Rice to orange-red-grained astaxanthin rice. The low-level expression of the endogenous *bhy* in the rice endosperm led to unsuccessful astaxanthin production, and, therefore, canthaxanthin was accumulated [82]. Production of astaxanthin and canthaxanthin has also been accomplished by introducing *crtZ* and *crtW* genes isolated from *Brevundimonas* sp. in *Arabidopsis thaliana* [103]*, Brassica napus* [83], *Solanum tuberosum* [4], *Nicotiana benthamiana* [80], *N. tabacum* [81], and *N. glauca* [104]. Soybean seeds (*Glycine max*) were also transformed with phytoene synthase gene from *Pantoea*, coupled with a β-carotene ketolase gene (*crtW* from *Brevundimonas* sp. or *bkt* from *H. pluvialis*) to produce ketocarotenoids [71]. These authors obtained transgenic seeds from both events that accumulated high amounts of canthaxanthin.

Despite reported accomplishments in generating transgenic systems producing canthaxanthin, it is still not clear how this complex pathway is regulated, and some efforts are underway to understand ketocarotenoids production. Hence, combinatorial expression of key genes from various sources and manipulation of gene expression levels are strategies being applied to unlock the metabolic network in order to improve the production of desired molecules [105].

As mentioned before, the enzymes involved in canthaxanthin biosynthesis can have distinct characteristics at the molecular level, and evolutionary analysis may be a powerful predicting tool to enhance carotenoid heterologous biosynthesis in a specific host on the grounds that enzymes from different species show distinct catalytic properties [93,105]. In search of an alternative process to produce ketocarotenoids efficiently, our group is using molecular farming for the production of these high value molecules. Our approach is the exploitation of cell cultures from model plants, since they offer tangible advantages when compared with traditional systems, in terms of cost reduction, safety, and growth in a controlled and confined environment [106,107]. We are also using photosynthetic microalgae as candidate molecular farming platforms for the production of high-value carotenoids, taking into consideration the evolution of the carotenoid biosynthesis pathway and all the various metabolic engineering outcomes from the work described in the previous section. Thus, similar methodologies are being implemented to partially extend the carotenoid pathway towards canthaxanthin and generate additional ketocarotenoid products in plant cell cultures and microalgae.

## 6. Concluding Remarks

During the past decade, metabolic engineering of eukaryotes has progressed remarkably for the production of several types of carotenoids towards their release into the market. Carotenoid engineering has great potential for the production and purification of specific carotenoids to be used as pharmaceuticals, feed/food supplements, and nutraceuticals [108]. From a biotechnological point of view, the ketocarotenoids astaxanthin and canthaxanthin are among the most important pigments as they are widely used in aquaculture and as feed and food additives [4,109].

Currently-used methodologies for the production of these molecules remain far from ideal, especially in light of a constant rise in consumer awareness and environmental sustainability concerns. Despite its high value and decades of research, as well as the relative flexibility to manipulate carotenogenic metabolic routes, the commercial production of canthaxanthin by biological systems remains challenging. The canthaxanthin market for personal care products and dietary supplements and additives is growing rapidly. Coupled with the feed/food industry, the canthaxanthin market size is predicted to expand over the coming years [34]. Therefore, the scope for further developments in the production of these molecules is high, with natural product extraction to compete with chemical synthesis [77,110]. Multiple biological systems have been explored as sustainable alternatives, including plants. Although plant-based platforms, such as cultured cells, are still far from being established as cost-effective alternative sources of canthaxanthin, they present a high potential for the production of ketocarotenoids. We envisage that future focus on these alternative producing platforms and a deeper knowledge of the regulation of the pathway, both at protein and genetic (including epigenetic) levels, will be key to boosting sustainable canthaxanthin production.

## Figures and Tables

**Table 1:** Reported organisms capable of de novo synthesis of canthaxanthin and other ketocarotenoids.

	Species	Major Ketocarotenoid	Other Carotenoids	Canthaxanthin Amount	Ref.
**Fungi**	*Aspergillus carbonarius*	Canthaxanthin (partially saturated)	-	20.6% DW	[37]
	*Cantharellus cinnabarinus*	Canthaxanthin	β-carotene	n.a.	[36]
	*Xanthophyllomyces dendrorhous*	Astaxanthin	β-carotene, canthaxanthin, echinenone, zeaxanthin	n.a.	[38,39]
**Microalgae**	*Asterarcys quadricellulare*	Astaxanthin	β-carotene, canthaxanthin, lutein	1.4% DW	[40]
	*Botryococcus braunii*	Canthaxanthin	β-carotene, echinenone, lutein, violaxanthin, zeaxanthin	46% DW	[41]
	*Chlorella emersonii*	Canthaxanthin	β-carotene, neoxanthin, violaxanthin	n.a.	[42]
	*Chlorella vulgaris*	Canthaxanthin	Astaxanthin, violaxanthin	n.a.	[43]
	*Chlorella zofingiensis*	Canthaxanthin	β-carotene, astaxanthin, lutein	25% TSC 2.1% of crude extract	[44,45]
	*Chlorococcum*	Adonixanthin	β-Carotene, astaxanthin, canthaxanthin, lutein	3.6–18.9% TC	[46]
	*Coelastrella striolata*	Canthaxanthin	β-carotene, astaxanthin	4.75% DW	[19]
	*Dactylococcus dissociatus*	Canthaxanthin	Lutein, neoxanthin, violaxanthin	1.20–3.9 mg/L of culture	[47]
	*Dictyococcus cinnabarinus*	Echinenone	β-carotene, canthaxanthin	n.a.	[48]
	*Haematococcus pluvialis*	Astaxanthin	β-carotene, canthaxanthin, echinenone, lutein, neoxanthin, violaxanthin, zeaxanthin	2.2–44.3% TC	[49,50,51]
	*Nannochloropsis*	Canthaxanthin	Antheraxanthin, astaxanthin, vaucheriaxanthin, violaxanthin, zeaxanthin	0.8–16.9% TC	[52]
	*Scenedesmus obliquus*	Astaxanthin	β-carotene, adonirubin, adonixanthin, antheraxanthin, canthaxanthin, echinenone, lutein, neoxanthin, violaxanthin	n.a.	[53]
**Bacteria**	*Bradyrhizobium* sp.	Canthaxanthin	Echinenone, spirilloxanthin	86.6% TC	[54]
	*Brevibacterium* sp.	Canthaxanthin	Echinenone	7.2 mg/L	[55]
	*Dietzia* sp.	Canthaxanthin	β-carotene, astaxanthin	90–92% TC	[56,57]
	*Gordonia jacobaea*	Canthaxanthin	β-carotene, astaxanthin	88% TC	[18]
	*Micrococcus roseus*	Canthaxanthin	α/β-carotene derivatives	85% TC	[58,59]
	*Paracoccus schoinia*	Adonixanthin diglucoside	α-carotene, α-cryptoxanthin, astaxanthin diglucoside, canthaxanthin, echinenone, zeaxanthin, zeaxanthin diglucoside	1% mol TC	[60]
**Archaea**	*Haloferax alexandrinus* TMT	Canthaxanthin	β-carotene	33.8% TC	[61,62]

Total carotenoids (TC), total secondary carotenoids (TSC), not available (n.a.), dry weight (DW).

**Table 2:** Production of ketocarotenoids in bacteria, fungi, and higher plants through metabolic engineering.

Species	Genes (Origin)	Major Carotenoid Accumulation	Ref.
*Escherichia coli*	*crtW* (*A. variabilis*)	β-carotene, β-cryptoxanthin, canthaxanthin	[77]
	*crtW* (*P.* N81106) *crtW* (*B.* sp. SD212)	Adonixanthin Canthaxanthin	[78]
*Nostoc* sp. PCC 7120	*crtO* (*N. flagelliforme*)	Echinenone, canthaxanthin	[79]
*Nicotiana benthamiana*	*crtZ* and *crtW* (*B.* sp. SD212)	Canthaxanthin, astaxanthin	[80]
*Nicotiana tabacum*	*crtZ* and *crtW* (*B.* sp. SD212)	Adonixanthin, canthaxanthin, astaxanthin, 4-ketoantheraxanthin	[81]
*Oryza sativa*	*psy1* (maize) *crtI* (*P. ananatis*) *bkt* (*H. pluvialis* or *C. reinhardtii*)	Astaxanthin, β-carotene, canthaxanthin	[82]
*Glycine max*	*crtB* (*P. ananatis*) *crtW* (*B*. sp. SD212) *bkt* (*H. pluvialis*)	β-carotene, canthaxanthin, astaxanthin	[71]
*Brassica napus*	*crtE* (*P. ananatis*) *crtW* (B. sp. SD212)	α-carotene, echinenone, lutein, canthaxanthin	[83]
*Daucus carota* L.	*bkt* (*H. pluvialis*)	Astaxanthin, adonirubin and canthaxanthin	[84]
*Solanum lycopersicum*	*bhy* (*H. pluvialis*) *bkt* (*C. reinhardtii*)	β-carotene, astaxanthin, adonirubin, canthaxanthin, echinenone	[85]
	*crtZ, crtW* (*B.* sp. SD212)	β-carotene, astaxanthin, phoenicoxanthin, adonixanthin, canthaxanthin, echinenone	[86]
*Lilium x formolongi*	*crtE, crtB, crtI, crtY* (*P. ananatis*) *crtZ, crtW* (*B.* sp. SD212) *idi* (*P.* sp. N81106)	Echinenone, canthaxanthin, 3-hydroxyechinenone, astaxanthin	[87]
*Mucor circinelloides*	*crtW* (*P.* sp. N81106)	β-carotene, echinenone, canthaxanthin	[88]

## Abbreviations

MEP (2-C-methyl-D-erythritol 4-phosphate), MVA (mevalonic acid), IPP (isopentenyl diphosphate), DMAPP (dimethylallyl diphosphate), BKT, CRTW and CRTO (β-carotene ketolase), CHY, HYDB, BCH, BHY and CRTZ (β-carotene hydroxylase), Nrf2 (nuclear factor erythroid 2-related factor 2), NF-ĸB (nuclear factor ĸB), CCD4 (carotenoid dioxygenase), MEGA X (Molecular Evolutionary Genetics Analysis), CDD (Conserved Domain Database), FA (fatty acid), NCBI (National Center for Biotechnology Information), FAD (Flavin adenine dinucleotide), CRTI (phytoene desaturase), HBFD (hydroxy-β-ring 4-dehydrogenase), SDH (saccharopine dehydrogenases), NADP (Nicotinamide adenine dinucleotide phosphate).
